# Enhanced anastomotic healing by Daikenchuto (TJ-100) in rats

**DOI:** 10.1038/s41598-018-19550-4

**Published:** 2018-01-18

**Authors:** Toshiaki Wada, Kenji Kawada, Kenjiro Hirai, Kosuke Toda, Masayoshi Iwamoto, Suguru Hasegawa, Yoshiharu Sakai

**Affiliations:** 1 Department of Surgery, Graduate School of Medicine, Kyoto University, Kyoto, Japan; 2 Department of Surgery, Otsu City Hospital, Otsu, Shiga Japan; 3 Department of Surgery, National Hospital Organization Himeji Medical Center, Himeji, Japan; 4 Department of Gastroenterological Surgery, Faculty of Medicine, Fukuoka University, Fukuoka, Japan

## Abstract

Daikenchuto (DKT), a traditional Japanese medicine, is widely used to treat various gastrointestinal disorders. This study aimed to investigate whether DKT could promote the anastomotic healing in a rat model. Pedicled colonic segments were made in left colon by ligation of the feeding arteries, and then intestinal continuity was restored. Colonic blood flow was analyzed by using ICG fluorescence imaging: Fmax, Tmax, T1/2, and Slope were calculated. Anastomotic leakage (AL) was found in 6 of 19 rats (31.6%) in the control group, whereas in 1 of 16 rats (6.2%) in the DKT group. The Fmax and Slope of DKT group were significantly higher than those of control group. DKT could promote the anastomotic healing, with the higher bursting pressure on postoperative day (POD) 2 and 5, the larger granulation thickness on POD 5, and neoangiogenesis on POD 5. Histological examination showed DKT exhibited a decreased inflammatory cell infiltration, enhanced fibroblast infiltration, and enhanced collagen density on POD 5. In the DKT group, the levels of TGFβ1 on POD 2 and VEGFα on POD5 were significantly higher, whereas the level of TNFα on POD 2 was significantly lower. Therefore, DKT could be effective for the prevention of AL following colorectal surgery.

## Introduction

Anastomotic leakage (AL) is the most dreaded complication after colorectal surgery and is known to cause high morbidity and mortality. The reported rate of AL following colorectal surgery remains approximately 10% worldwide^1,2^. The pathophysiology of AL remains unclear, although numerous studies have been conducted on animals and humans. Despite the multifactorial etiology of AL, vascular perfusion is considered critical to anastomotic healing. Therefore, objective and accurate measurements of the intestinal perfusion are necessary to reduce the AL rate. Although several methods, including doppler technology and oxygen spectroscopy, have been proposed to assess intestinal perfusion, they are still experimental and cannot be used in regular clinical applications. In recent years, near-infrared (NIR) fluorescence technology with indocyanine green (ICG) has emerged as the most promising method to enable an accurate evaluation of the intestinal perfusion^3–5^. ICG is a cyanine dye and its fluorescence imaging is based on the principal that plasma protein-bound ICG emits light with a wavelength of 830 nm when illuminated by NIR light of 760–780 nm. Therefore, it can reveal the presence of an ischemic perfusion before performing the anastomosis.

Daikenchuto (DKT) is a traditional Japanese medicine (Kampo) that is a mixture of extract powders from dried Japanese pepper, processed ginger, ginseng radix, and maltose powder. DKT is reported to have three major effects: (i) improvement of intestinal movement, (ii) up-regulation of colonic blood flow, and (iii) activation of anti-inflammatory effect^6–9^. The anti-inflammatory effect is attributable to the down-regulation of cyclooxygenase-2 and the up-regulation of endogenous adrenomedullin^6,7^. An increase in intestinal blood flow is due to the up-regulation of calcitonin gene-related peptide^8^. Accelerated intestinal motility is due to modulation of intestinal contraction and relaxation by the release of acetylcholine, nitric oxide, and other excitatory neurotransmitters^10^. DKT is widely used for the treatment of various gastrointestinal disorders, including postoperative ileus and ischemic intestinal disorders^11^. Moreover, a randomized, double-blind study on healthy humans in the United States indicated that DKT significantly accelerated intestinal transit in the small intestine and ascending colon^12^. Several randomized controlled trials on the patients with postoperative ileus, Crohn’s disease, functional constipation and irritable bowel syndrome are currently on-going in the United States and Japan.

We hypothesized that DKT may prevent AL via its above-mentioned effects. Therefore, the aim of this study was to investigate whether DKT could promote the anastomotic healing following colorectal surgery in an AL rat model.

## Materials and Methods

### Animals

Wistar rats (8 weeks; Japan SLC, Shizuoka, Japan) were used for the animal experiments. DKT was provided from Tsumura & Co (Tokyo, Japan). All animal experiments were performed in accordance with the International Guiding Principles for Biomedical Research Involving Animals, and this study protocol was approved by the Animal Care and Use Committee of Kyoto University.

### Experimental Protocol

Thirty five rats were divided into the two groups: the control group (n = 19) and the DKT group (n = 16). The rats were sacrificed on postoperative day (POD) 2 or POD 5. In the DKT group, DKT (300 mg/kg) was administered orally 1 hour before the operation and one time per day from POD 1 to POD 2 (n = 8) or POD 5 (n = 8). In the control group, distilled water was administered 1 hour before the operation and one time per day from POD 1 to POD 2 (n = 10) or POD 5 (n = 9).

### Surgical Procedures

**Figure 1:**
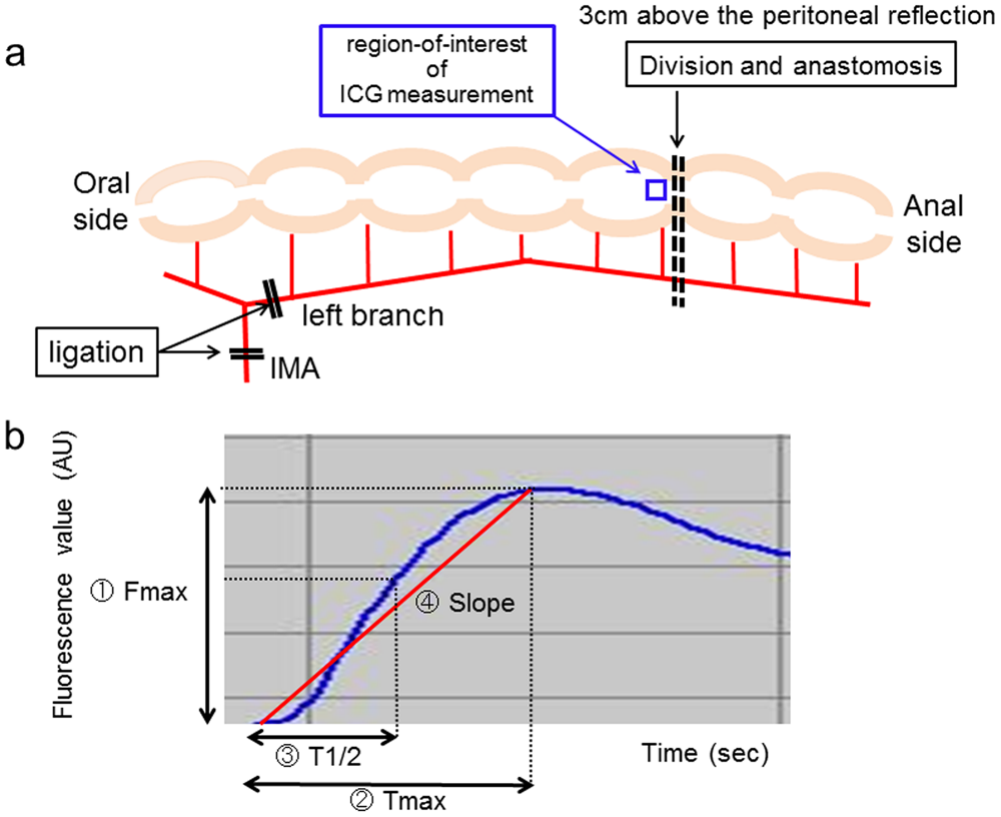
(**a**) A schematic representation of the model used in this study. Inferior mesenteric artery (IMA) and its left branch were sequentially ligated, and then the distal colon was transected 3 cm above the peritoneal reflection. ICG fluorescence measurement was performed at the distal end of the pedicled segment (blue square point). After ICG fluorescence measurement, intestinal continuity was restored by an end-to-end anastomosis. (**b**) Time curve of ICG fluorescence intensity. Fmax (the magnitude of intensity from the onset of ICG to the maximum intensity), Tmax (time from the onset of ICG to the maximum intensity), T1/2 (time required to reach the half of maximum from the onset of ICG), and Slope (Fmax/Tmax).

After a 3-cm midline laparotomy was performed, the pedicle segment of the colon was created by sequentially ligating the inferior mesenteric artery (IMA) and its left branch (Fig. 1a), which was modified from the ischemia colon model previously reported by Posma *et al*.^13^. Before bowel resection, the NIR camera system (PDE-neo system; Hamamatsu Photonics K.K., Hamamatsu, Japan) was fixed 15 cm away from the pedicled ischemic colon for the analysis of colonic blood flow, taking the focal distance of this system into consideration (Supplementary Fig. 1a). The distal colon was transected 3 cm above the peritoneal reflection. After ICG fluorescence imaging movie was recorded, intestinal continuity was restored by an end-to-end anastomosis (8 stitches of interrupted sutures using 6–0 PDS). The anastomotic site was evaluated on POD 2 (n = 18) or POD 5 (n = 17). At sacrifice on POD 2 or POD 5, the anastomotic site was graded from 1 to 3, as previously described^14^; (1) no, AL (2) small abscess at the anastomotic site <1 cm^3^, (3) large (>1 cm^3^) abscess at the anastomotic site, or (4) complete dehiscence with peritonitis/death due to faecal peritonitis. Grades 2, 3 and 4 were defined as  AL. All surgical procedures were performed by a board-certified surgeon (T.W.).

### Measurement of Colonic Blood Flow

During the operation, the colonic blood flow was analyzed using ICG fluorescence imaging. We used a PDE-neo system and a luminance analysis software (ROIs; Hamamatsu Photonics K.K.) for the quantitative evaluation of ICG fluorescence (Supplementary Fig. 1). The stored video files were analyzed retrospectively by using the software, ROIs^15–17^. In the quantitative measurement, the region-of-interest was positioned at the transection site (Fig. 1a). A time-dependent curve of fluorescence intensity was constructed, and the following 4 parameters were calculated: Fmax (the magnitude of intensity from the onset of ICG to the maximum intensity), Tmax (time from the onset of ICG to the maximum intensity), T1/2 (time required to reach the half of maximum from the onset of ICG), and Slope (Fmax/Tmax) (Fig. 1b).

### Measurement of Bursting Pressure

On POD 2 or POD 5, a 5-cm segment of colon including the anastomotic site with adherent organs was resected en bloc. A diameter 3 mm catheter was placed into the oral side of the descending colon, and both sides of the colon were ligated to close the lumen. The catheter was connected to an infusion syringe and a manometer (Handy Manometer PG-100B; Copal Electronics, Tokyo, Japan). The bursting pressure of the anastomosis was measured as the intraluminal pressure at which air leakage from the anastomosis was initially observed.

### Histopathlogical Assessment

After measuring the bursting pressure, samples containing the anastomotic site were fixed in a 4 wt% formaldehyde solution and embedded in paraffin. Sections (5 μm) were cut and stained with hematoxylin and eosin (H&E). For the histological grading scale, infiltration of inflammatory cells (i.e., lymphocytes, plasma cells, and polymorphonuclear leucocytes) or fibroblasts were evaluated at the anastomotic site using a modified 0-to-4 Ehrlich and Hunt numerical scale: 0 = no evidence, 1 = occasional evidence, 2 = light scattering, 3 = abundant evidence and 4 = confluent cells or fibers^18^. The sections were viewed systemically at ×100 magnification. Regarding collagen density, collagen deposition at the anastomotic site was scored using the same scale. The sections were stained with anti-type III collagen antibody (SouthernBiotech, Birmingham, AL;1330–01) and scanned systemically at ×200 magnification. All assessments were conducted by two researchers (T.W., and K.H.) blinded to the experimental groups.

### Thickness of Granulation

The thickness of the granulation tissue at the anastomotic site was measured. The H&E sections were viewed systemically at ×40 magnification. Granulation tissues were morphologically identified as tissues containing collagen fibers, fibroblasts, microvessels and inflammatory cells in the center part of the anastomotic site. The measurement was performed for three sections and the average was calculated.

### Neovascularization at the Anastomotic Site

The sections were stained with anti-von Willebrand factor (vWF) (Abcam, Cambridge, UK; ab6994) and scanned systemically at ×400 magnification. The vWF-positive blood vessels were counted at the anastomotic site (3 fields (1 mm^2^) analyzed per sample). The measurement was performed for three sections and the average was calculated.

### Measurement of Inflammatory Cytokines by Quantitative Reverse Transcription Polymerase Chain Reaction (RT-PCR)

On POD 2 (n = 7) or POD 5 (n = 8), samples containing the well-healed anastomotic site were resected and stocked in a refrigerator of −80 degrees. Total RNAs were extracted and reverse transcription was carried out. The resulting cDNA was quantified using StepOnePlus^TM^ Real-Time PCR System (Applied Biosystems) and THUNDERBIRD SYBR qPCR Mix (TOYOBO). Primer sequences for GAPDH, IL6, TNFα, IFNγ, TGFβ1, IL10, IL1β and VEGFα were listed in Supplementary Table 1.

### Statistical Analysis

All values were expressed as the means ± standard deviation (SD). Continuous variables were determined by Student’s *t*-test. The Fisher’s exact test was used for comparison and analysis of categorial variables. All analyses were two-sided and a *P* value of <0.05 was considered statistically significant in all analyses. Statistical analyses were conducted with JMP Pro software, 11.0.0 (SAS Insititute INC, NC).

### Compliance with ethical standards

The experimental protocol was approved by the Animal Care and Use Committee of Kyoto University.

## Results

### Anastomotic Leakage (AL)

In total, AL was found in 6 of 19 rats (31.6%) in the control group at sacrifice, whereas it was in 1 of 16 rats (6.2%) in the DKT group (*P* = 0.09). In the control group, AL was occurred in 4 and 2 rats on POD 2 and POD 5, respectively. In the DKT group, AL was found in 1 rat on POD 2.

### Colonic Blood Flow measured by ICG Fluorescence Imaging

**Figure 2:**
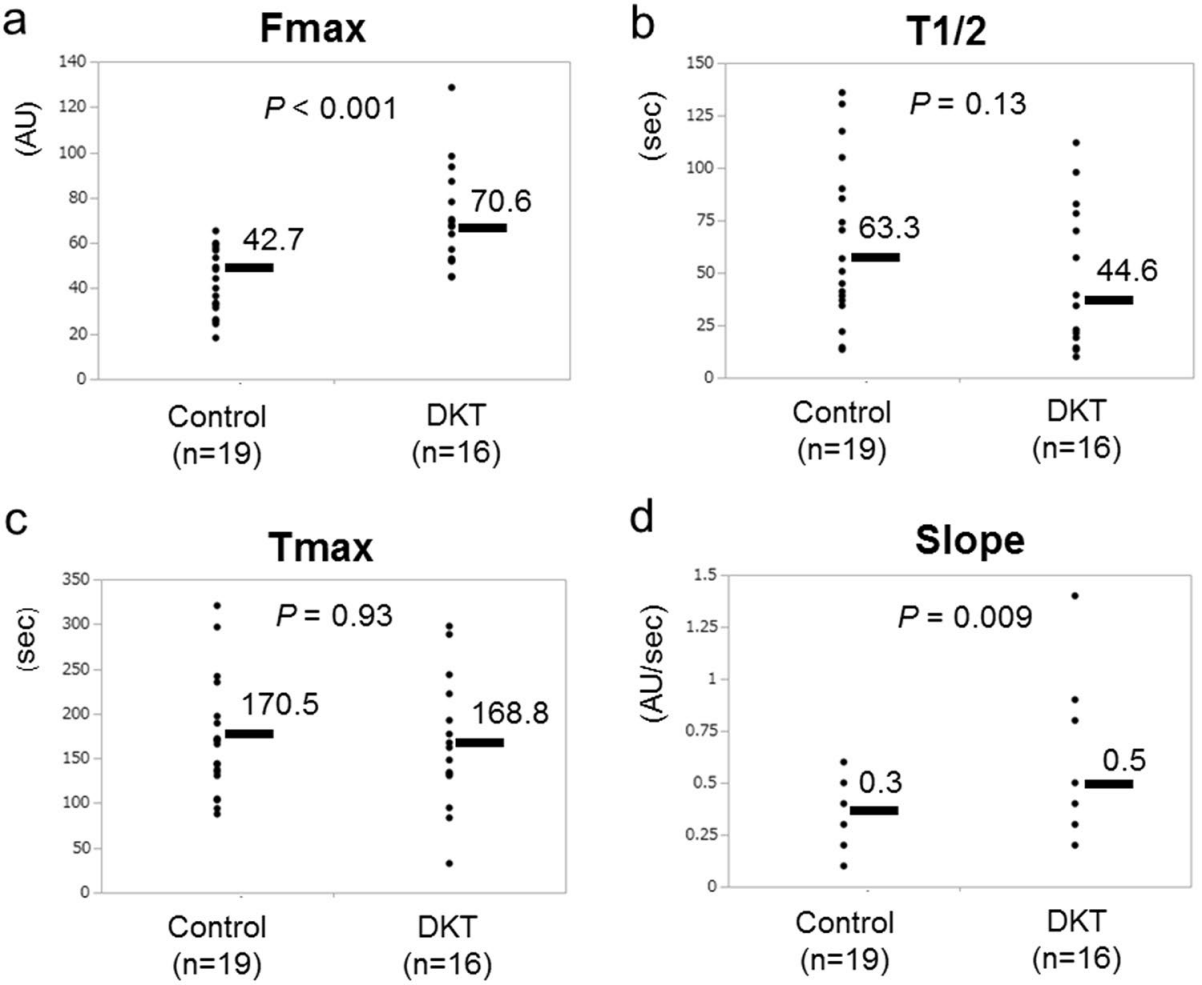
Comparison of ICG fluorescence-related parameters between the control group and DKT group. Fmax (**a**), T1/2 (**b**), Tmax (**c**) and Slope (**d**). Means bars. Student’s t test.

To measure the colonic blood flow, intraoperative ICG fluorescence imaging was obtained in all cases. After ligation of the IMA and its left branch, the colonic blood flow of the pedicle segment was analyzed by using ICG fluorescence imaging. The fluorescence intensity of the pedicle segment was gradually decreasing to the anal side, but the demarcation line determined by ICG fluorescence was not found in this model (Supplementary Fig. 1b). With the recorded video images, we retrospectively created a time-fluorescence intensity curve at the point of bowel transection (i.e., 3 cm above the peritoneal reflection) using a software, ROIs: Fmax, Tmax, T1/2, and Slope were measured (Fig. 2 and Supplementary Fig. 2). Regarding the Fmax and Slope, there was a significant difference between the control group and DKT group. The Fmax of the DKT group was significantly higher than that of the control group (70.6 ± 4.5 vs. 42.7 ± 4.2 arbitrary units (AU); *P* < 0.001). The Slope of the DKT group was significantly higher than that of the control group (0.5 ± 0.05 vs. 0.3 ± 0.05 AU/sec; *P* = 0.009). Meanwhile, there was no significant difference between the two groups regarding the T1/2 or Tmax.

### Anastomotic Bursting Pressure

**Figure 3:**
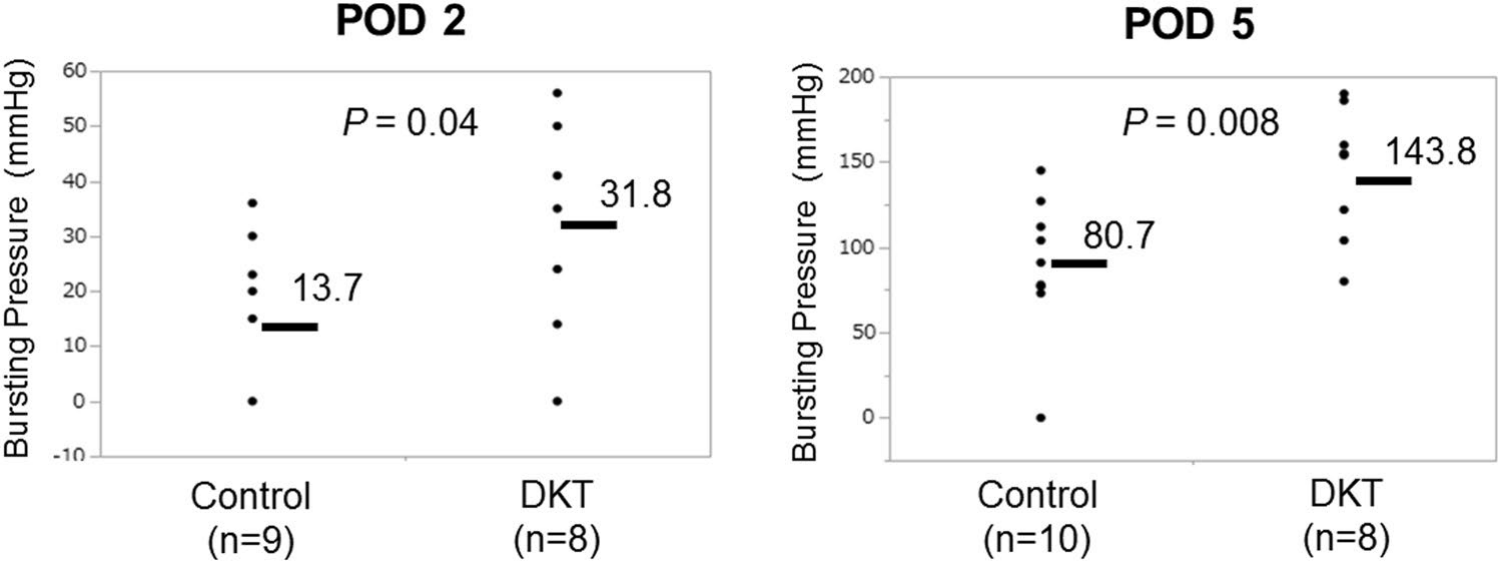
The bursting pressure (mmHg) was measured on POD 2 (left) and 5 (right). Values are expressed as means bar.

The anastomotic bursting pressure significantly increased from POD 2 to POD 5 (Fig. 3). On POD 2, the bursting pressure of the DKT group was significantly higher than that of the control group (31.8 ± 5.8 vs. 13.7 ± 5.4 mmHg; *P* = 0.04). Similarly, on POD 5, the bursting pressure of the DKT group was also significantly higher than that of the control group (143.8 ± 15.7 vs. 80.7 ± 15.7 mmHg; *P* = 0.008).

**Figure 4:**
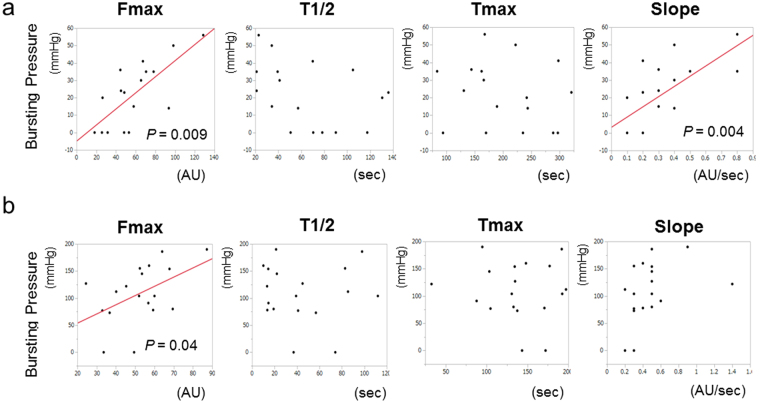
Relationship between the bursting pressure and ICG fluorescence-related 4 parameters. POD 2 (**a**) and POD 5 (**b**).

Furthermore, we examined whether there was a correlation between colonic blood flow and bursting pressure. Fmax and Slope were significantly correlated with the bursting pressure on POD 2 (*P* = 0.009 and 0.004, respectively; Fig. 4a). In addition, Fmax was also significantly correlated with the bursting pressure on POD 5 (*P* = 0.04; Fig. 4b). Meanwhile, T1/2 and Tmax were not correlated with the bursting pressure.

**Figure 5:**
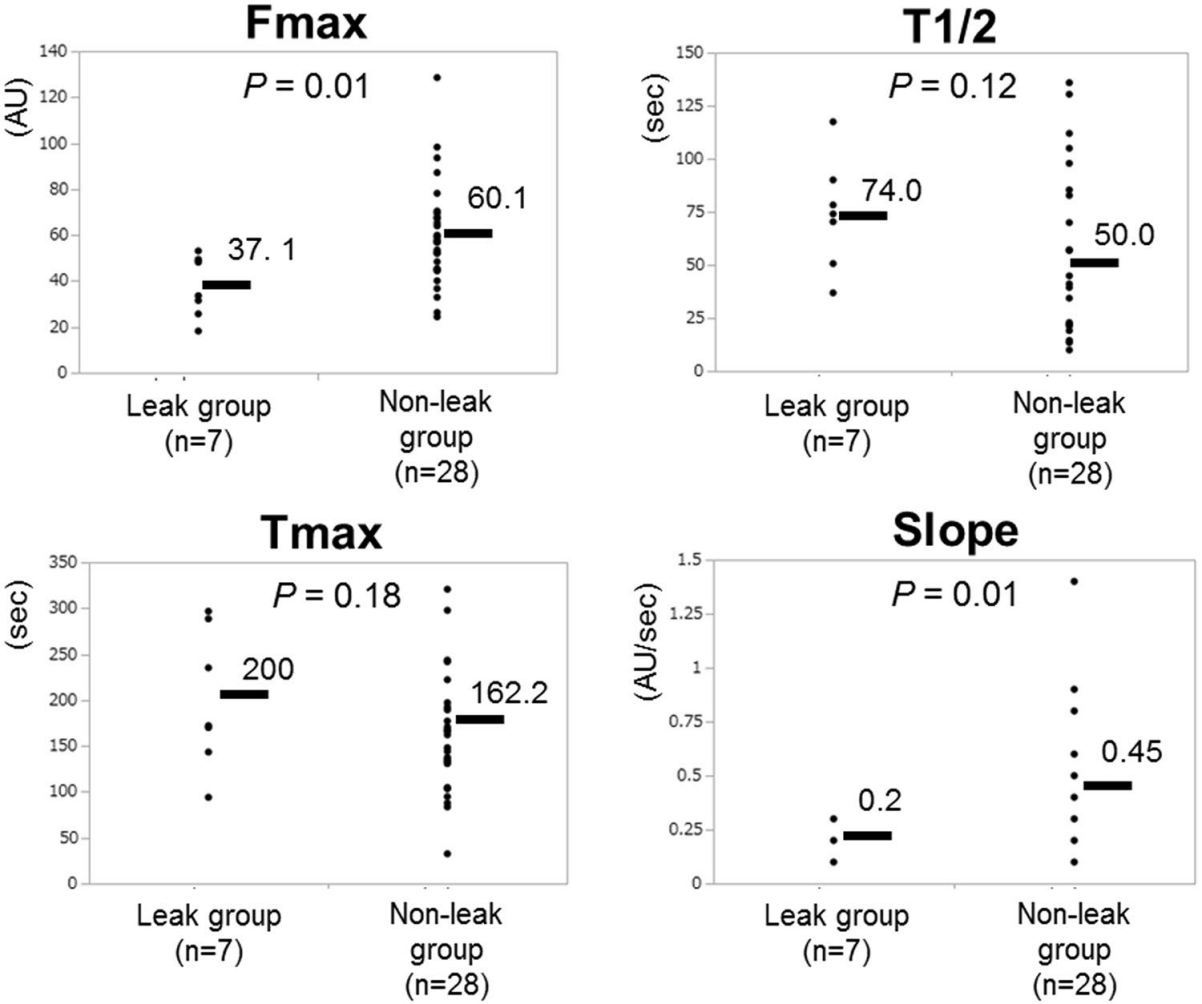
Comparison of ICG fluorescence-related 4 parameters between the leak group (n = 7) and non-leak group (n = 28).

We also examined whether there was a correlation between colonic blood flow and AL (Fig. 5). Fmax and Slope of the non-leakage group (n = 7) were significantly higher than those of the leakage group (n = 28) (*P* = 0.01 and 0.01, respectively). Meanwhile, Tmax and T1/2 were not significantly correlated with AL.

### Histopathological Evaluation

**Figure 6:**
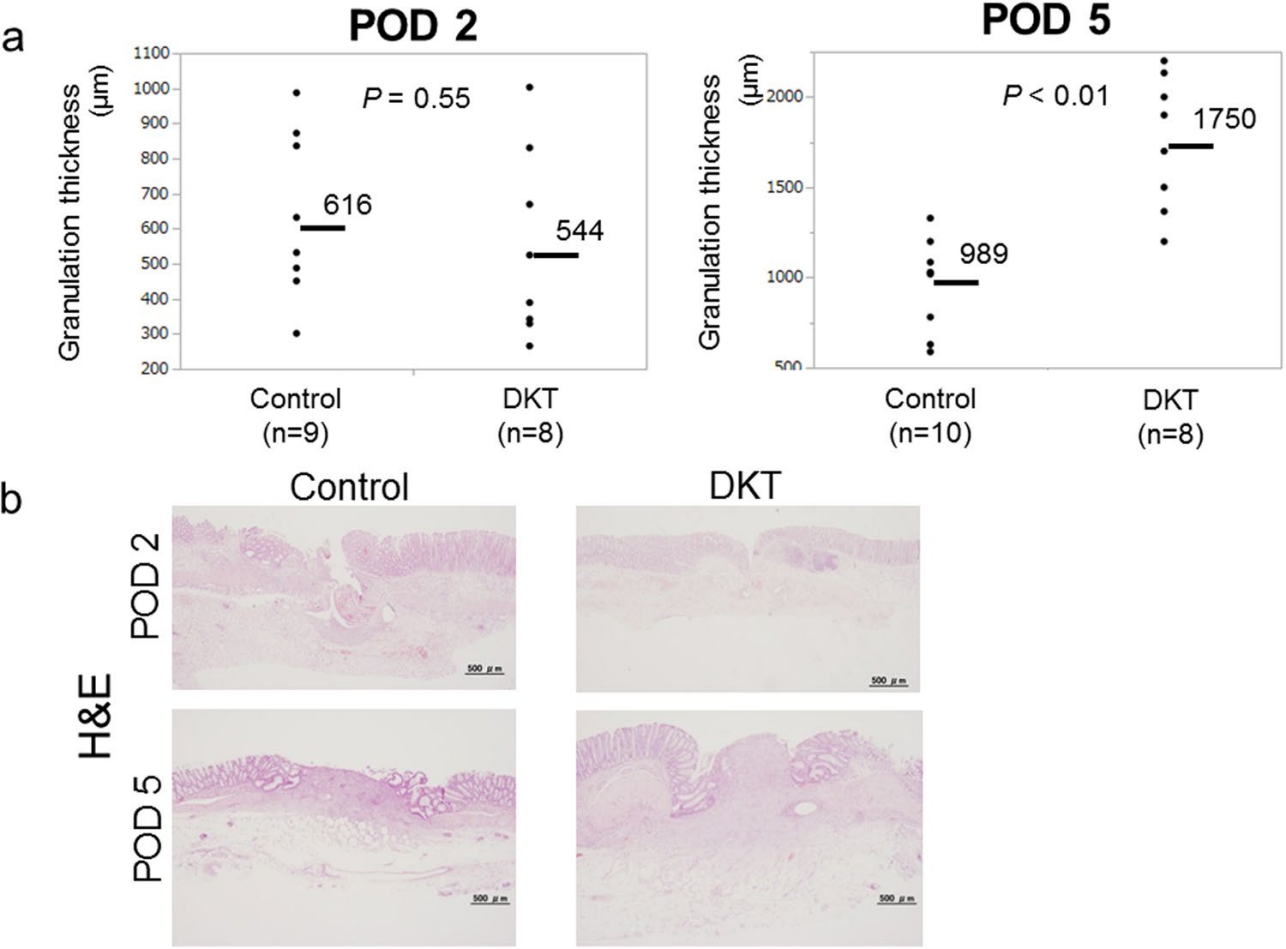
(**a**) Comparison of granulation thickness between the control group and DKT group on POD 2 (left) and 5 (right). Means bars. Student’s t test. (**b**) Histological sections of the anastomosed site on POD 2 (upper) and 5 (lower) with hematoxylin and eosin (H&E). ×40.

Figure 6 shows the thickness of the granulation tissue. A significant increase in granulation thickness at the anastomotic site was identified on POD 5 for the DKT group compared with the control group (1750 ± 108.2 vs. 989.1 ± 96.7 μm; *P* < 0.01). However, no significant difference was found on POD 2 between the two groups (544.2 ± 87.8 vs. 616 ± 82.8 μm; *P* = 0.55).

The levels of inflammatory cell infiltration, fibroblast infiltration, and collagen density at the anastomotic site were evaluated according to the modified Ehrlich and Hunt numerical scale^18^ (Supplementary Table 2 and Supplementary Fig. 3). Inflammatory cell infiltration was marked on POD 2, while fibroblast infiltration and collagen density were marked on POD 5. On POD 2, there were no significant differences between the DKT group and the control group. Meanwhile, on POD 5, the DKT group exhibited decreased inflammatory cell infiltration, enhanced fibroblast infiltration, and enhanced collagen density, although the differences were not statistically significant (*P* = 0.12, 0.06, and 0.15, respectively).

**Figure 7:**
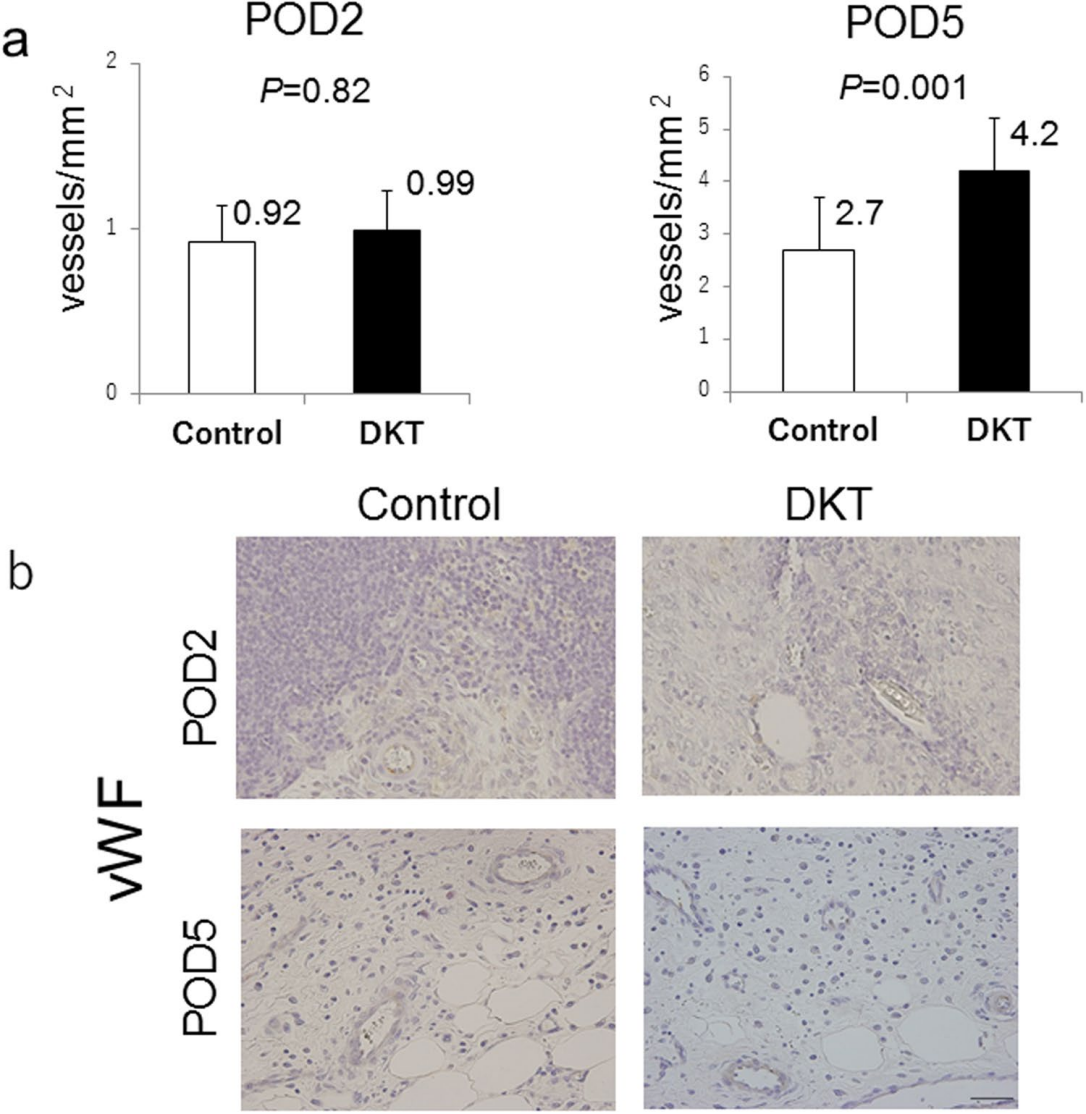
(**a**) Comparison of neovascularization at the anastomotic site between the control group and DKT group on POD 2 (left) and 5 (right). Means bars. Student’s t test. (**b**) Histological sections of the anastomosed site on POD 2 (upper) and 5 (lower), stained with vWF. ×400.

Figure 7 shows the number of vWF-positive blood vessels at the anastomotic site (3 fields (1 mm^2^)). On POD 2, there were no significant differences between the two groups. Meanwhile, on POD 5, the number of vWF-positive blood vessels in the DKT group was significantly higher than that in the control group (4.2 ± 0.28 vs. 2.7 ± 0.25 vessels/mm^2^; *P* = 0.001)

### Inflammatory Cytokines at the Anastomotic Site

**Figure 8:**
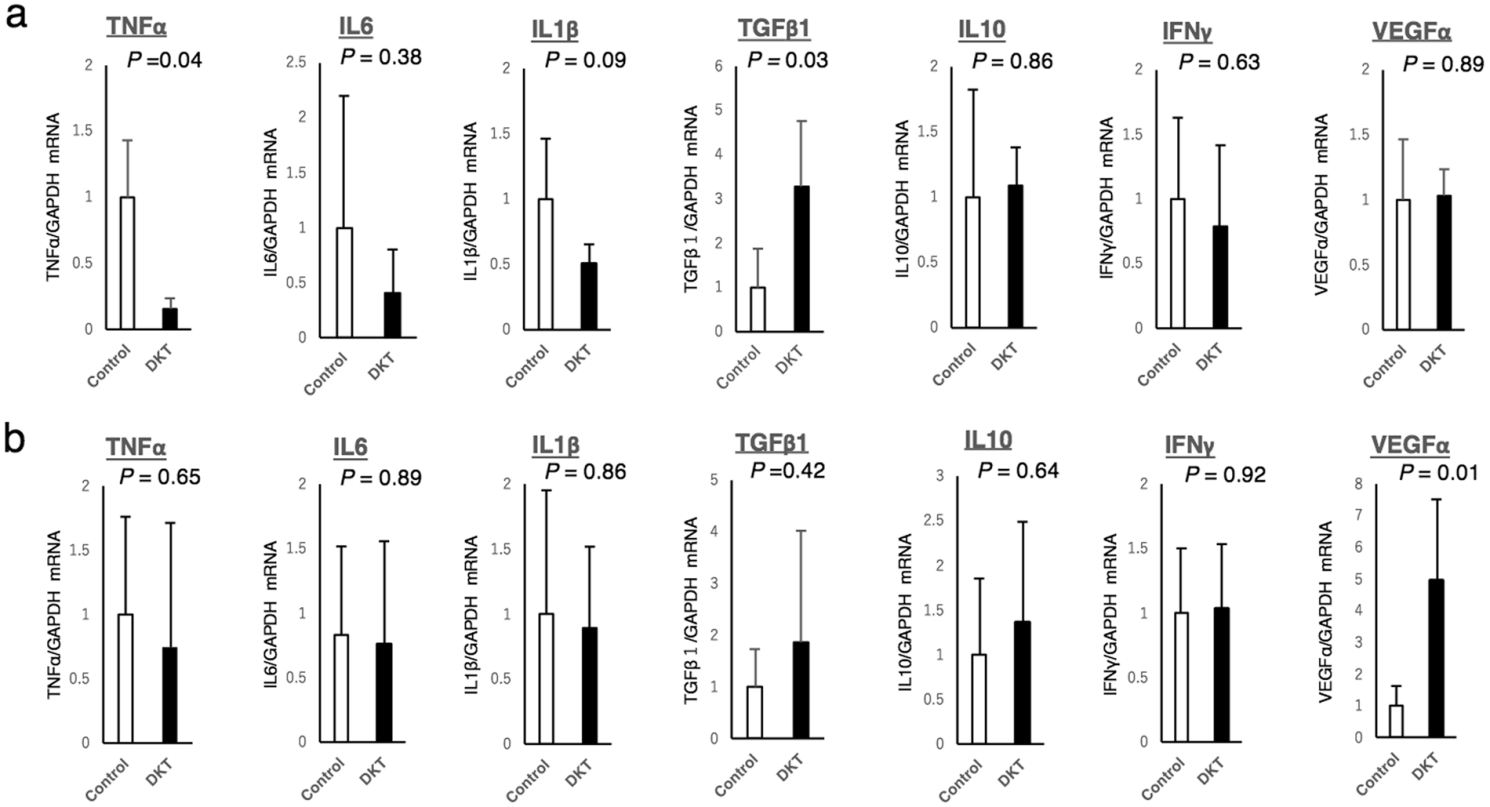
Expression levels of inflammatory cytokines (TNFα, IL6, IL1β, TGFβ1, IL10, IFNγ and VEGFα) measured by quantitative RT-PCR. POD 2 (**a**) and POD 5 (**b**).

Figure 8 shows the mRNA expression levels of inflammatory cytokines (TNFα, IL6, IL1β, TGFβ1, IL10, IFNγ, and VEGFα) measured by quantitative RT-PCR at the well-healed anastomotic site. In the DKT group, on POD 2, TGFβ1 was significantly increased (*P* = 0.03), while TNFα was significantly decreased (*P* = 0.04). On POD 5, only VEGFα was significantly increased in the DKT group (*P* = 0.01). Meanwhile, there was no significant difference between the two groups as to the other cytokines.

## Discussion

DKT has been clinically used to treat various gastrointestinal diseases, including postoperative ileus, abdominal bloating and cold sensation in the abdomen. DKT has specific functions such as improving intestinal movement, increasing colonic blood flow, and suppressing inflammation. Kono *et al*. reported that DKT could increase intestinal perfusion in rats by up-regulating calcitonin gene-related peptide (CGRP) and adrenomedullin (ADM)^8,19^. Therefore, we examined whether these effects of DKT could result in the promotion of anastomotic healing following colorectal surgery in an AL rat model. In the present study, Fmax and Slope were significantly higher in the DKT group than in the control group (Fig. 2). Importantly, DKT significantly improved the bursting pressure on both POD 2 and 5 (Fig. 3), the granulation thickness at the anastomotic site on POD 5 (Fig. 6), neoangiogenesis at the anastomotic site on POD 5 (Fig. 7), and the histopathological inflammation scores on POD 5 (Supplementary Table 2). In addition, we examined the effect of DKT on several inflammatory cytokines in this model. In the DKT group, the levels of TGFβ1 on POD 2 and VEGFα on POD5 were significantly higher, whereas that of TNFα on POD 2 was significantly lower (Fig. 8). TGFβ1 plays a role in wound healing and scar formation. VEGFα induces angiogenesis and endothelial cell proliferation, which results in the regulation of vasculogenesis. TNFα is involved in systemic inflammation and one of the cytokines that make up the acute phase reaction. Meanwhile, there was no significant difference between the two groups as to IL6, IL1β, IL10, and IFNγ (Fig. 8). Previous studies have shown that DKT promotes a variety of growth factors and cytokines, such as TNFα, IFNγ, and IL1β, that can accelerate the healing of organ injury. In a TNBS-induced colitis model, Kono *et al*. reported that DKT significantly decreased the expressions of TNFα and IFNγ in the colonic mucosa^20^. In a CPT11-induced intestinal injury model, Chikakiyo *et al*. reported that DKT significantly decreased the expressions of IL1β and IFNγ in the small intestine^21^. In a T cell transfer-induced colitis model, Iwasa *et al*. reported that DKT significantly decreased IL-17 expression, although the expression levels of TNFα and IFNγ did not change^22^. In a postoperative ileus model, Endo *et al*. reported that DKT significantly decreased TNF-α expression in the small intestine^23^. In a bacterial translocation model, Yoshikawa *et al*. reported that DKT prevented bacterial translocation by maintaining intestinal barrier integrity via anti-apoptotic and anti-inflammatory effects through the reduced expressions of inflammatory cytokines, including IFNγ and TNFα^24^.

Recent evidence suggests a role of intestinal bacteria in the pathophysiology of AL. In an AL rat model, *Enterococcus faecalis* contributes to the pathogenesis of AL through collagen degradation and matrix metalloproteinase-9 activation, and that the elimination of *Enterococcus faecalis* through direct topical antibiotics could prevent AL^25^. In a rat model of low anterior resection, the combination of preoperative RT and intestinal inoculation with *Pseudomonas aeruginosa* resulted in a higher AL rate^26^. In addition, it was reported that butyrate, a short-chain fatty acid, produced by microbiota strengthed colonic anastomosis in a rat model^27^. Notably, Yoshikawa *et al*. reported that DKT prevented the reduction of microbial diversity in the inflammatory intestine of rat, which indicates the new anti-inflammatory effect of DKT through gut micobiome^28^. However, clinical implications for these findings are lacking. A large retrospective cohort has recently reported that combined preoperative mechanical bowel preparation with oral antibiotics significantly reduces AL and surgical site infection following colorectal surgery^29^. It remains to be elucidated whether eliminating intestinal bacteria by antibiotics or promoting the growth of certain species by probiotics could improve anastomotic healing.

ICG fluorescence imaging has been used to evaluate blood flow perfusion in several surgical fields such as reconstructive surgery, cardiovascular surgery, and gastrointestinal surgery^30–32^. Although an NIR camera system can reveal ICG images of blood flow in real time, whether ICG fluorescence imaging is available for preventing AL in the colorectal surgery remains controversial^4,5,33,34^. In the present study, we investigated colonic blood flow by using ICG fluorescence imaging in an AL animal model. By using luminance analysis software, we quantified the colonic blood flow as four parameters: Fmax, T1/2, Tmax, and Slope. This is the first report to investigate the colonic blood flow by comparing these four parameters in an AL animal model. We found that Fmax and Slope of the colonic blood flow were significantly increased by DKT administration, although T1/2 and Tmax were not (Fig. 2). Posma *et al*. reported that ligation of one feeding artery or three arteries in the colon of rats could reduce the mean Slope to 59% or 26%, respectively, and that there was no significant correlation between Slope and the bursting pressure on POD 2 and 5^13^. Meanwhile, in the present study, Fmax and Slope were significantly correlated with the bursting pressure on POD 2 (Fig. 4a). In addition, Fmax was also significantly correlated with the bursting pressure on POD 5. The differences in animal model, NIR system, and manometer used to measure bursting pressure might account for the differences between the present study and Posma’s previous study. In an ischemic bowel animal model, Diana *et al*. reported that lactate level of the 25% Slope area was significantly higher than that of the 75% Slope area, and that the histopathological inflammation score was higher in the 25% Slope area than in the 75% Slope area^35^. In a porcine model, Nerup *et al*. reported that the regional blood perfusion of the stomach was significantly associated with both Slope and Fmax^36^. In a small bowel strangulation model, Matsui *et al*. reported that qualitative and/or quantitative metrics, including Fmax, could be predictive of histological grade and animal survival^37^. In the present study, Fmax and Slope were significantly associated with AL (Fig. 5), which indicates that Fmax and Slope could be predictive of AL. In a clinical setting, we previously reported that preoperative chemotherapy and anticoagulation therapy were significantly correlated to poor intestinal perfusion when evaluated by ICG fluorescence imaging^38^. In addition, we have recently reported that Fmax and Slope could be predictive of AL from a retrospective analysis of 112 patients who underwent laparoscopic surgery for left-sided colorectal cancers^17^, which is consistent with the results of the present animal study.

The present study has some limitations. A systematic review has recently reported that the animal research to investigate AL needs to be improved before results can be applied into the clinical setting^39^. In addition to the variety of animal models, a wide range of endpoints are used, although the most frequently used surrogate marker of AL in animal models is the bursting pressure of the anastomotic site. Notably, an international consensus statement was reached on several recommendations for the use of animal models for research on AL in the lower gastrointestinal tract^40^.

In conclusion, this study has provided the first evidence of the potential effectiveness of DKT in the prevention of AL following colorectal surgery. The efficacy of DKT for anastomotic healing may be attributable to its capacity to increase blood flow as well as anti-inflammatory effect.

## Electronic supplementary material

Supplementary information

## References

[1] Kang CY (2013). Risk factors for anastomotic leakage after anterior resection for rectal cancer. JAMA Surg..

[2] Matsubara N (2014). Mortality after common rectal surgery in Japan: a study on low anterior resection from a newly established nationwide large-scale clinical database. Dis Colon Rectum..

[3] Kudszus S, Roesel C, Schachtrupp A, Hoer JJ (2010). Intraoperative laser fluorescence angiography in colorectal surgery: a noninvasive analysis to reduce the rate of anastomotic leakage. Langenbecks Arch Surg..

[4] Hellan M, Spinoglio G, Pigazzi A, Lagares-Garcia JA (2014). The influence of fluorescence imaging on the location of bowel transection during robotic left-sided colorectal surgery. Surg Endosc..

[5] Jafari MD (2015). Perfusion assessment in laparoscopic left-sided/anterior resection (PILLAR II): a multi-institutional study. J Am Coll Surg..

[6] Hayakawa T (1999). Effects of Dai-kenchu-to on intestinal obstruction following laparotomy. J Smooth Muscle Res..

[7] Kono T (2011). Daikenchuto (TU-100) ameliorates colon microvascular dysfunction via endogenous adrenomedullin in Crohn’s disease rat model. J Gastroenterol..

[8] Kono T (2008). Colonic vascular conductance increased by Daikenchuto via calcitonin gene-related peptide and receptor-activity modifying protein 1. J Surg Res..

[9] Fukuda H (2006). The herbal medicine, Dai-Kenchu-to, accelerates delayed gastrointestinal transit after the operation in rats. J Surg Res..

[10] Kito Y, Suzuki H (2006). Effects of Dai-kenchu-to on spontaneous activity in the mouse small intestine. J Smooth Muscle Res..

[11] Kono T, Kanematsu T, Kitajima M (2009). Exodus of Kampo, traditional Japanese medicine, from the complementary and alternative medicines: is it time yet?. Surgery..

[12] Manabe N (2010). Effect of daikenchuto (TU-100) on gastrointestinal and colonic transit in humans. Am J Physiol Gastrointest Liver Physiol..

[13] Posma LA (2010). Reduction of oxygenation and blood flow in pedicled bowel segments in the rat and its consequences for anastomotic healing. Dis Colon Rectum..

[14] Bosmans J (2017). Comparison of three different application routes of butyrate to improve colonic anastomotic strength in rats. Int J Colorectal Dis..

[15] Terasaki H (2013). A quantitative method for evaluating local perfusion using indocyanine green fluorescence imaging. Ann Vasc Surg..

[16] Kawaguchi Y (2015). Usefulness of Intraoperative Real-Time Tissue Elastography During Laparoscopic Hepatectomy. J Am Coll Surg..

[17] Wada T. *et al*. ICG fluorescence imaging for quantitative evaluation of colonic perfusion in laparoscopic colorectal surgery. *Surg Endosc*. 2017 Mar 9. [Epub ahead of print]. 10.1007/s00464-017-5475-3 28281123

[18] Ehrlich HP, Trarver H, Hunt TK (1973). Effects of vitamin A and glucocorticoids upon inflammation and collagen synthesis. Ann Surg..

[19] Kono T (2013). Epithelial transient receptor potential ankyrin 1 (TRPA1)-dependent adrenomedullin upregulates blood flow in rat small intestine. Am J Physiol Gastrointest Liver Physiol..

[20] Kono T (2010). Anti-colitis and -adhesion effects of daikenchuto via endogenous adrenomedullin enhancement in Crohn’s disease mouse model. J Crohn’s Colitis..

[21] Chikakiyo M (2012). Kampo medicine “Dai-kenchu-to” prevents CPT-11-induced small-intestinal injury in rats. Surg Today..

[22] Iwasa T (2012). Feeding administration of Daikenchuto suppresses colitis induced by naive CD4+ T cell transfer into SCID mice. Dig Dis Sci..

[23] Endo M (2014). Daikenchuto, a traditional Japanese herbal medicine, ameliorates postoperative ileus by anti-inflammatory action through nicotinic acetylcholine receptors. J Gastroenterol..

[24] Yoshikawa K (2008). Kampo medicine “Dai-kenchu-to” prevents bacterial translocation in rats. Dig Dis Sci..

[25] Shogan BD (2015). Collagen degradation and MMP9 activation by Enterococcus faecalis contribute to intestinal anastomotic leak. Sci Transl Med..

[26] Olivas AD (2012). Intestinal tissues induce an SNP mutation in Pseudomonas aeruginosa that enhances its virulence: possible role in anastomotic leak. PLoS One..

[27] Bloemen JG (2010). Butyrate enemas improve intestinal anastomotic strength in a rat model. Dis Colon Rectum..

[28] Yoshikawa K (2013). Effect of Kampo medicine “Dai-kenchu-to” on microbiome in the intestine of the rats with fast stress. J Med Invest..

[29] Kiran RP, Murray AC, Chiuzan C, Estrada D, Forde K (2015). Combined preoperative mechanical bowel preparation with oral antibiotics significantly reduces surgical site infection, anastomotic leak, and ileus after colorectal surgery. Ann Surg..

[30] Nachiappan S (2014). Intraoperative assessment of colorectal anastomotic integrity: a systematic review. Surg Endosc..

[31] Still J (1999). Evaluation of the circulation of reconstructive flaps using laser-induced fluorescence of indocyanine green. Ann Plast Surg..

[32] Waseda K (2009). Intraoperative fluorescence imaging system for on-site assessment of off-pump coronary artery bypass graft. JACC Cardiovasc Imaging..

[33] Boni L (2016). Indocyanine green-enhanced fluorescence to assess bowel perfusion during laparoscopiccolorectal resection. Surg Endosc..

[34] Kin C, Vo H, Welton L, Welton M (2015). Equivocal effect of intraoperative fluorescence angiography on colorectal anastomotic leaks. Dis Colon Rectum..

[35] Diana M (2015). Intraoperative fluorescence-based enhanced reality laparoscopic real-time imaging to assess bowel perfusion at the anastomotic site in an experimental model. Br J Surg..

[36] Nerup N (2017). Quantification of fluorescence angiography in a porcine model. Langenbecks Arch Surg..

[37] Matsui A, Winer JH, Laurence RG, Frangioni JV (2011). Predicting the survival of experimental ischaemic small bowel using intraoperative near-infrared fluorescence angiography. Br J Surg..

[38] Kawada K (2017). Evaluation of intestinal perfusion by ICG fluorescence imaging in laparoscopic colorectal surgery with DST anastomosis. Surg Endosc..

[39] Yauw S, Wever K, Hoesseini A, Ritskes-Hoitinga M, van Goor H (2015). Systematic review of experimental studies on intestinal anastomosis. Br J Surg..

[40] Bosmans J (2016). International consensus statement regarding the use of animal models for research on anastomoses in the lower gastrointestinal tract. Int J Colorectal Dis..

