# Supplementary material for: Enhanced anastomotic healing by Daikenchuto (TJ-100) in rats
Source: Sci Rep. 2018 Jan 18;8:1091. doi: 10.1038/s41598-018-19550-4 (PMC5773623; doi:10.1038/s41598-018-19550-4)
Supplement: Supplementary file 1 — Supplementary information [file 41598_2018_19550_MOESM1_ESM.pdf]

Enhanced anastomotic healing by Daikenchuto (TJ-100) in rats

Toshiaki Wada[1], Kenji Kawada[1*], Kenjiro Hirai[1,2] Kosuke Toda[1], Masayoshi Iwamoto[3], Suguru Hasegawa[4], Yoshiharu Sakai[1].

Department of Surgery, Graduate School of Medicine, Kyoto University, Kyoto, Japan[1].
Department of Surgery, Otsu City Hospital, Otsu, Shiga, Japan[2].
Department of Surgery, National Hospital Organization Himeji Medical Center, Himeji, Japan[3].
Department of Gastroenterological Surgery, Faculty of Medicine, Fukuoka University, Fukuoka, Japan[4].

Supplementary Fig. 1

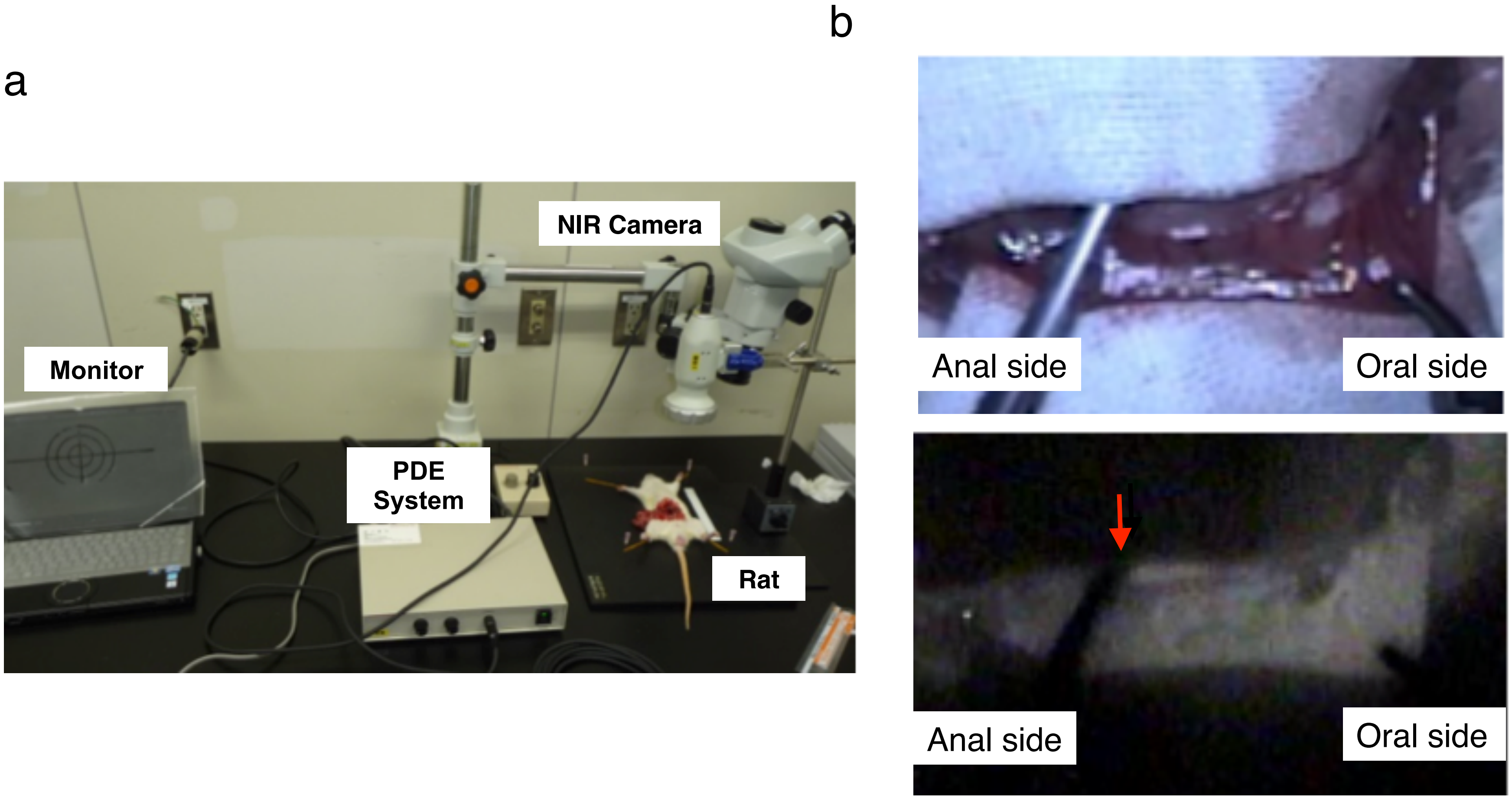

Supplementary Fig. 2

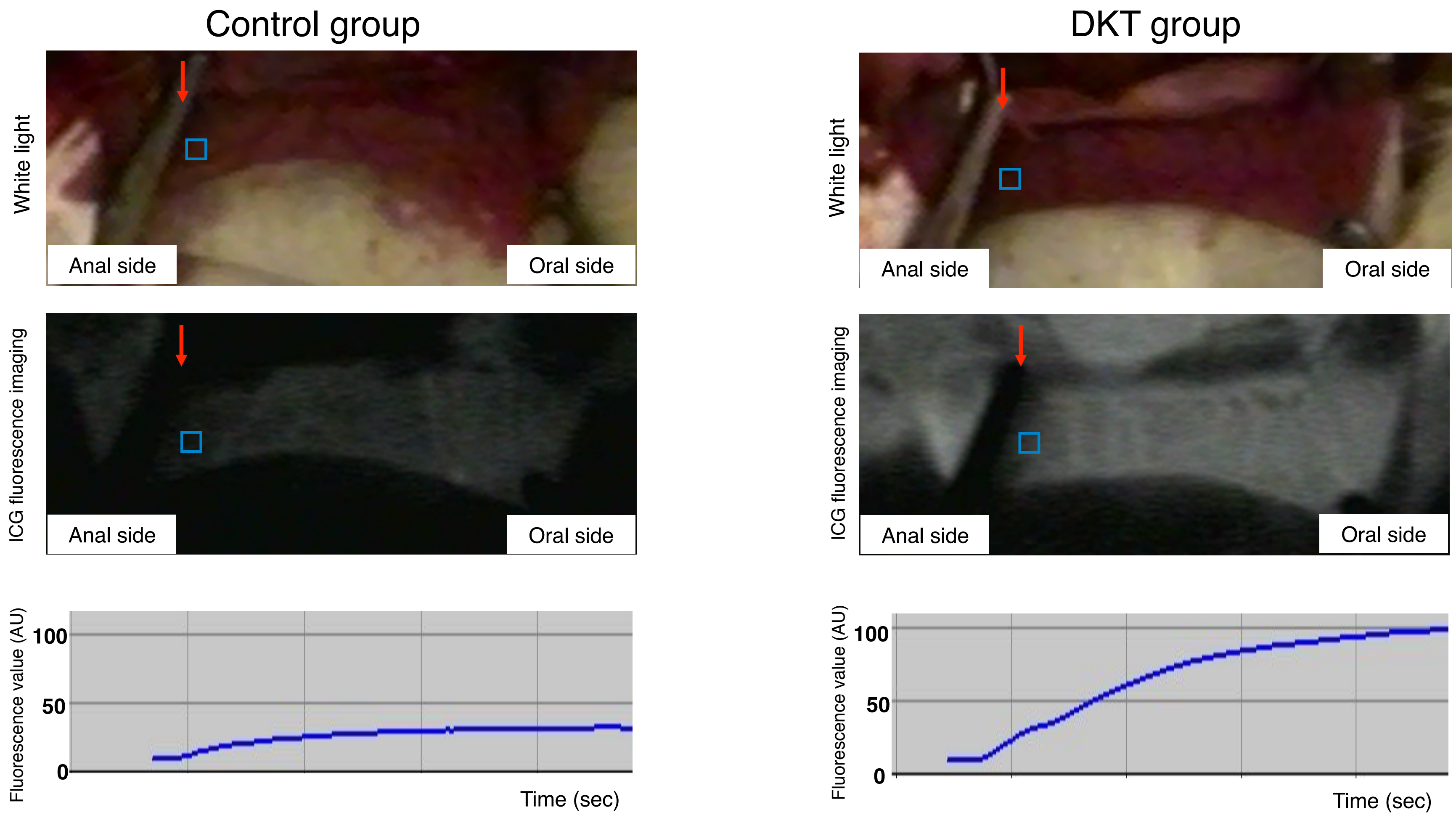

Supplementary Fig. 3

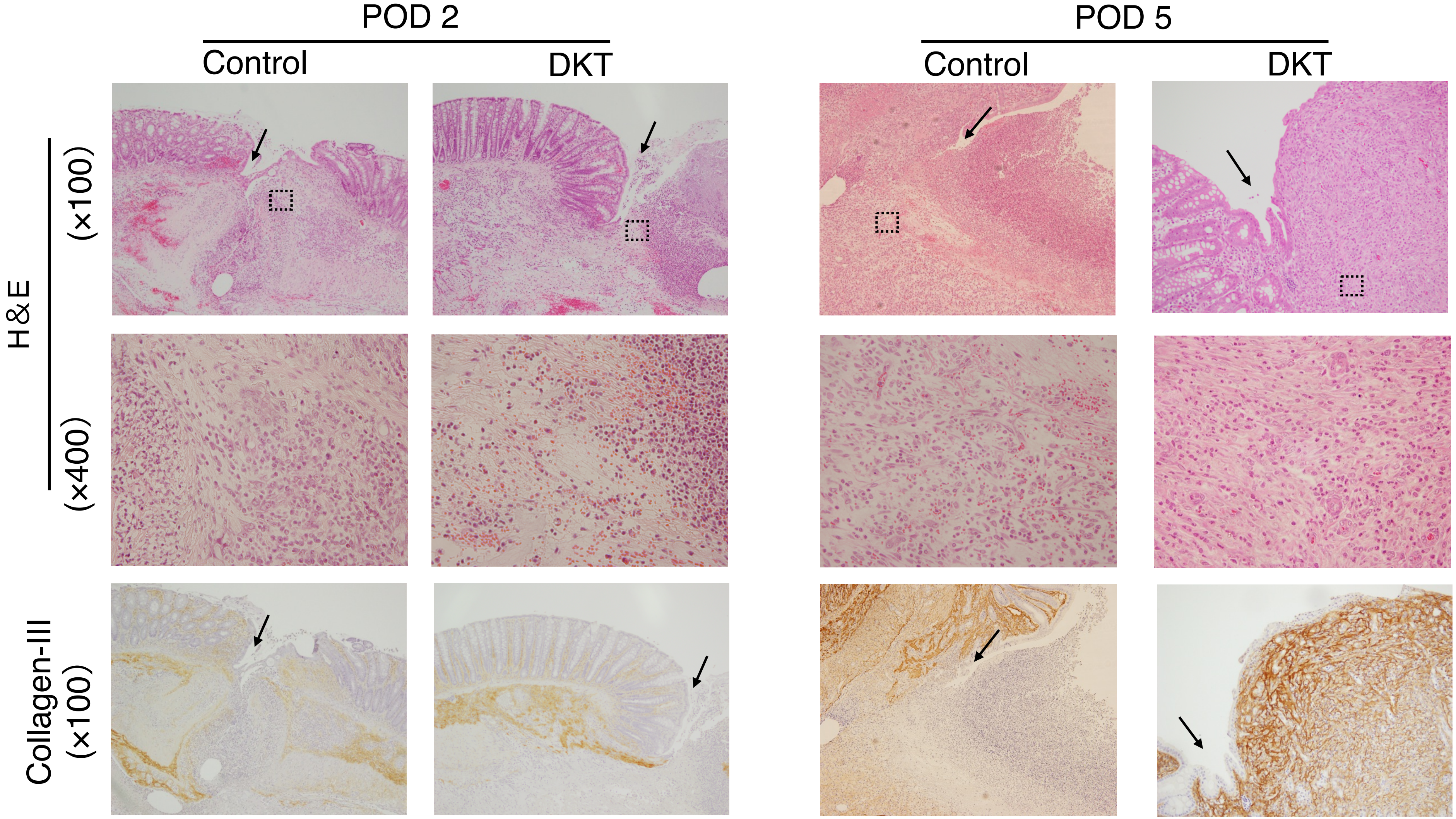

Supplementary Table 1

Primer sequence for qRT-PCR

GAPDH	forward	TTGTGATGGGTGTGAACCAC
GAPDH	reverse	GGATGCAGGGATGATGTTCT
IL6	forward	AGTTGCCTTCTTGGGACTGA
IL6	reverse	CAGAATTGCCATTGCACAAC
TNFα	forward	ACTCCCAGAAAAGCAAGCAA
TNFα	reverse	CGAGCAGGAATGAGAAGAGG
IFNγ	forward	GCCCTCTCTGGCTGTTACTG
IFNγ	reverse	CTGATGGCCTGGTTGTCTTT
TGF-β1	forward	TGAGTGGCTGTCTTTTGACG
TGF-β1	reverse	TGGGACTGATCCCATTGATT
IL10	forward	GAATTCCCTGGGAGAGAAGC
IL10	reverse	CAAGGAGTTGCTCCCGTTAG
IL-1β	forward	AGTCTGCACAGTTCCCCAAC
IL-1β	reverse	GAATGTGCCACGGTTTTCTT
VEGFα	forward	CAAGATCCGCAGACGTGTAA
VEGFα	reverse	GACTGTTCTGTCGACGGTGA

Supplementary Table 2

	POD 2			POD 5		
	Control (n=9)	DKT (n=8)	*P* value	Control (n=10)	DKT (n=8)	*P* value
Inflammatory cell infiltration	2.77 ± 0.15	2.62 ± 0.16	0.52	2.30 ± 0.17	1.87 ± 0.19	0.12
Fibroblast infiltration	1.22 ± 0.15	1.37 ± 0.16	0.52	2.10 ± 0.17	2.62 ± 0.19	0.06
Collagen density	0.66 ± 0.14	0.87 ± 0.15	0.34	2.40 ± 0.15	2.75 ± 0.17	0.15

Legends to Supplementary Figures and Tables

Supplementary Figure 1. (a) A picture shows operating setting. The NIR camera system was fixed 15 cm away from the rats. ICG fluorescence imaging of colonic blood flow was displayed on a monitor in real-time. (b) The distal colon was shown under the white/visible light (upper) and ICG fluorescence imaging (bottom). Red arrows indicate the point where distal colon was divided and then anastomosed.

Supplementary Figure 2. Representative photos of the DKT group and control group are shown under the white/visible light (upper) and ICG fluorescence imaging (middle). ICG fluorescence measurement was performed at the distal end of the pedicled segment (blue square point). Red arrows indicate the point where distal colon was divided and then anastomosed. Time curve of ICG fluorescence intensity are shown (bottom).

Supplementary Figure 3. Histological sections of the anastomosed sites on POD 2 and 5. Hematoxylin and eosin (H&E): ×100 (upper). The dotted square areas are shown with higher magnifications (×400; middle). Collagen type-III staining: ×100 (bottom). Black arrows indicate the anastomotic sites.

Supplementary Table 1. Primer sequence for qRT-PCR.

Supplementary Table 2. Comparison of histological grading scale at the anastomotic site on POD 2 and POD 5.
